# Optimization of Dual‐Bacteriophage Magnetic Nanoprobe for the Detection of *Staphylococcus aureus* and *Pseudomonas aeruginosa*

**DOI:** 10.1002/mabi.70237

**Published:** 2026-08-08

**Authors:** Jun‐Ping Zhang, Hsin‐Yi Lin

**Affiliations:** ^1^ Department of Chemical Engineering and Biotechnology National Taipei University of Technology Taipei Taiwan

**Keywords:** bacteriophage‐based analysis, dual‐bacterial detection, magnetic separation, *Pseudomonas aeruginosa*, *Staphylococcus aureus*, superparamagnetic nanoprobe

## Abstract

Due to the high host specificity of bacteriophages, a single‐phage superparamagnetic nanoprobe can only detect one bacterial species at a time. In this study, we developed a series of dual‐phage magnetic nanoprobes and performed a series of evaluations to identify the optimal formulation. Phages specific to *Staphylococcus aureus* and *Pseudomonas aeruginosa* were chemically conjugated to superparamagnetic iron oxide nanoparticles. The lytic activity of each phage was assessed individually and in combination. Six single‐phage nanoprobes and nine dual‐phage nanoprobes with different phage loading levels were prepared. The lowest detectable bacterial concentration and the number of bacteria captured by each probe were subsequently determined. We developed a dual‐phage superparamagnetic nanoprobe with high sensitivity and a detection limit of 10^1^ CFU/mL for either bacterium. The optimal probe contained 10^7^ PFU/mL of each phage. Increasing the phage loading beyond this level reduced bacterial capture efficiency. Additionally, the number of P‐phages that could be conjugated to nanoparticles decreased when a high concentration of S‐phages was present. No cross‐infection between phages and non‐target bacteria was observed, minimizing the risk of false‐positive detection.

## Graphical abstract

The optimal densities of two bacteriophages on magnetic nanoparticles are determined in order to make magnetic nanoprobes. These nanoprobes can bind bacteria in 10 min and be quickly retrieved from aqueous samples for later analysis. The nanoprobes are capable of capturing *Staphylococcus aureus* and *Pseudomonas aeruginosa* with concentrations as low as 10^1^ CFU/mL in aqueous solutions.

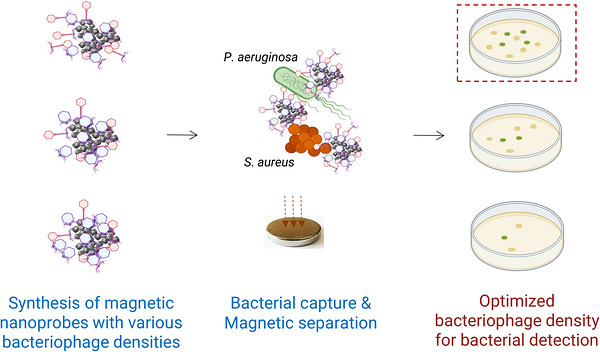

## 1 Introduction

Bacteriophages (phages) are viruses that specifically infect bacterial hosts. After infection, they replicate within the host cell and eventually lyse it to release progeny phages. This high host specificity enables the development of phage‐based superparamagnetic nanoprobes capable of selectively detecting pathogenic bacteria commonly found in contaminated water sources at concentrations ranging from 10^2^ to 10^4^ PFU/mL [1, 2, 3].

Different phage‐ and antibody‐based technologies have been developed to detect bacteria. Their sensitivities varied from 10^0^–10^5^ CFU/mL and detection time range from less than 1 h to 16 h (Table 1 by Shiue et al. 2024) [2]. Iron oxide nanoparticles have been used as a carrier for bacteriophages to capture bacteria from biological samples. Their superparamagnetism allows for easy and fast separation using a magnet.

**TABLE 1:** Concentrations of S‐phage and P‐phage in 1‐mL solutions used to prepare the phage‐linked probes, along with the corresponding probe notation. The numbers following “P” and “S” indicate the logarithmic order of phage concentration (PFU/mL).

P (PFU) S (PFU)	0	10^5^	10^7^	10^9^
0	—	P5	P7	P9
10^5^	S5	P5S5	P5S7	P5S9
10^7^	S7	P7S5	P7S7	P7S9
10^9^	S9	P9S5	P9S7	P9S9

In a previous study, we synthesized highly sensitive phage‐conjugated superparamagnetic iron oxide nanoparticles that specifically detect Shiga toxin‐producing *Escherichia coli* O157:H7 [2]. Both the bacteria and nanoprobes were removed from the aqueous phase using simple magnetic separation. We demonstrated that 10 mg of the phage‐linked nanoprobe in a 1 mL sample could detect as few as 10^2^ CFU/mL of *E. coli* O157:H7. However, because a phage probe only recognizes its specific bacterial host, a single‐phage nanoprobe can detect only one pathogen at a time. Thus, detecting two pathogens simultaneously would require doubling the amount of single‐phage probes. An alternative strategy is to engineer a nanoprobe carrying two phage types, provided that the dual‐phage formulation can achieve sensitivity comparable to each individual phage probe. To the best of our knowledge, no prior studies have systematically evaluated the detection performance of iron oxide nanoprobes conjugated with two or more phages.

In this study, we developed a dual‐phage nanoprobe targeting *Staphylococcus aureus* (*S. aureus*) and *Pseudomonas aeruginosa* (*P. aeru*). These two pathogens are among the most common bacteria co‐isolated from chronically infected wounds [4]. *P. aeru* is a Gram‐negative bacterium with a prevalence of 7.1%–7.3% among healthcare‐associated infections [5], while *S. aureus* is a Gram‐positive bacterium and one of the most frequent causes of infectious diseases, contributing significantly to morbidity and mortality in adults [6]. Early detection of *P. aeru* and *S. aureus* is essential for timely infection management and improved patient outcomes.

Phages specific to *P. aeru* and *S. aureus* were conjugated to iron oxide nanoparticles to create a series of dual‐phage nanoprobes with varying phage loading ratios. One aim of this study was to determine whether the presence of one phage on the nanoparticle surface affects the binding affinity of the other. We also sought to identify optimal phage loading concentrations that maximize bacterial detection sensitivity. Finally, we investigated whether cross‐infection between phages and non‐target bacteria occurs, which could lead to false‐positive results.

To achieve these objectives, we quantified the maximum number of phages that could be conjugated to 10 mg of iron oxide nanoparticles, and we measured the bacterial capture efficiency of dual‐phage nanoprobes with different phage ratios. We also determined the zeta potential of both nanoprobes and bacterial cells, as surface charge influences nanoprobe–bacteria binding efficiency [7]. These results enabled us to evaluate whether a dual‐phage nanoprobe can detect specific bacterial pathogens as effectively as single‐phage probes and to identify optimal phage loading conditions for dual‐target detection.

## 2 Materials and Methods

### 2.1 Phage Purification

Wild‐type phages were isolated from wastewater using the double‐agar plaque overlay method [8, 9]. Phages purified from *S. aureus* (BCRC 10781, Bioresource Collection and Research Center [BCRC], Taiwan) and *P. aeru* (BCRC 11633) will be hereinafter referred to as S‐phage and P‐phage, respectively.

Phage concentrations were measured using the double‐agar plaque assay combined with the drop method [9]. The average concentration of both phages in the stock solutions was approximately 4 × 10^9^ PFU/mL.

### 2.2 Specificity

To confirm that each purified phage infected only its specific host strain, 10 µL of each phage solution was dropped onto double‐agar plates containing either *S. aureus* or *P. aeru*. Plaque formation was examined after 24 h. The presence of plaques indicated successful infection of the corresponding host, while absence of plaques confirmed no cross‐infection.

### 2.3 Phage Lytic Activity in a Mixture

To determine whether the presence of one phage affects the lytic activity of the other, phage solutions were diluted and mixed according to the combinations listed in Table 1. “P5” refers to a solution containing P‐phage at approximately 10^5^ PFU/mL, and “S7” refers to a solution containing S‐phage at approximately 10^7^ PFU/mL. For example, a P5S7 mixture contained P‐phage at 10^5^ PFU/mL and S‐phage at 10^7^ PFU/mL. A total of nine groups were tested: P5, P7, P9, S5, S7, S9, P9S5, P7S7, and P5S9. The lytic activity of each phage or phage mixture was evaluated by counting plaques formed using the drop method described in Section 2.1.

### 2.4 Preparation and Characterization of Phage‐Linked Nanoprobes

Chitosan‐coated iron oxide nanoparticles (cNP) were prepared according to previously published methods [2, 10]. Freshly synthesized cNP (10 mg dry weight) was then activated using EDC and NHS as described earlier [2]. After rinsing with sterile water, the NHS/EDC‐activated cNP was mixed with 1 mL of the phage solutions listed in Table 1 and incubated at 4°C for 16 h. The resulting phage‐grafted cNP constituted the nanoprobes: six single‐phage probes and nine dual‐phage probes. The probes were magnetically separated and stored in 1 mL of 0.1% (w/w) bovine serum albumin (Sigma‐Aldrich) [11] at 4°C overnight.

In this study, a single‐phage nanoprobe prepared from the P5 solution is referred to as the P5 probe, while the S7 probe corresponds to the nanoprobe prepared from the S7 solution. Dual‐phage nanoprobes follow the same notation; for example, the P5S7 probe was prepared using the P5S7 phage mixture.

#### 2.4.1 Morphology of Phage, Nanoprobes, and Bacteria‐Bound Nanoprobes

Transmission electron microscopy (TEM, 200 kV, HT‐7000, Hitachi, Japan) was performed to visualize phages and nanoprobes before and after bacterial binding. To prepare samples, 20 µL of suspension was placed on a TEM grid for 2 min. Lens paper was used to remove excess liquid. Samples were stained with phosphotungstic acid for 90 s. Excess liquid was removed and samples were allowed to air dry. TEM images were analyzed to measure individual cNP sizes using ImageJ (NIH) (*n* = 100).

#### 2.4.2 Fourier‐Transform Infrared Spectroscopy (FTIR)

FTIR analysis was conducted to confirm the presence of chitosan on the cNP surface. FTIR spectra were obtained for pure iron (II, III) oxide nanoparticles (NP; Sigma‐Aldrich, MO, USA), cNP, and chitosan. Samples were scanned using an FTIR instrument (Spectrum 3, Perkin Elmer, USA) in attenuated total reflection (ATR) mode from 4000 to 600 cm^−1^, with four scans collected at a spectral resolution of 4 cm^−1^. Prior to sample measurement, an instrumental background scan was recorded against ambient air. Background normalization and baseline corrections were applied to the raw signal using the native software (PerkinElmer Spectrum IR, version 10).

#### 2.4.3 Dynamic Light Scattering (DLS)

Surface zeta potentials of bacterial hosts, phages, and nanoprobes were measured to evaluate surface charge changes after phage grafting, as surface charge may influence nanoprobe‐bacteria interactions. NP and cNP samples (1 mg/mL) were sonicated for 1 h prior to measurement using a Zetasizer (Nano‐ZS90, Malvern, UK).

Bacterial suspensions were centrifuged at 7000 g (Kubota centrifuge model 7780, Japan), and pellets were resuspended in water to a concentration of10^8^ CFU/mL. Phage suspensions were centrifuged at 20 000 g and resuspended in water to 10^9^ PFU/mL, and nanoprobes were rinsed with water and resuspended to 0.1 mg/mL before measurement.

### 2.5 Quantification of Phages Loaded on Single‐ and Dual‐Phage Nanoprobes

After 10 mg of EDC/NHS‐treated cNP reacted with the phage solutions for 16 h (Section 2.4), the nanoprobes were magnetically separated and the remaining number of free phages (N) in the supernatant was quantified using the drop method described in Section 2.1. The percentage of phages loaded onto 10 mg of cNP was calculated as: [(N_0_‐N)/N_0_] × 100%, where N_0_ is the initial number of phages in the solution.

### 2.6 Bacterial Capture and Detection Limit of Single‐ and Dual‐Phage Nanoprobes

To determine bacterial capture efficiency, 10 mg of nanoprobes were added to 1 mL of bacterial suspensions in phosphate buffered saline (PBS, pH 7) containing 10^1^ CFU, 10^2^ CFU and 10^3^ CFU. An overnight bacterial culture suspension with O.D. 600 at 0.4 contained bacterial concentrations in the 10^8^ CFU/mL range. Serial dilution was made to obtain bacterial concentrations needed for the test. The actual number of bacteria in the three dilutions were determined by the plating method (N_0_) in each test. After 10 min of mixing of the probes in the bacterial suspension, bacteria‐bound probes were magnetically separated, rinsed with PBS, and transferred into 500 µL of water. The suspension was vortexed for 30 s to detach bacteria from the probes, which were then magnetically removed. The number of remaining bacteria (N) in the solution was quantified using the pour plate method. The percentage of bacteria captured by the probes was calculated as (N_0_−N)/N_0_ × 100%. The detection limit of a probe was defined as the lowest bacterial concentration (CFU/mL) at which bacteria could be consistently recovered. A lower detection limit indicates higher sensitivity.

The specificities of the single‐phage nanoprobes were tested. The S‐probes were added to *P. aeru* suspension (10^4^ CFU/mL), and the P‐probes were added to 10^4^ CFU/mL *S. aureus* suspension. Ten minutes later the number of bacteria captured by the probes were measured by the pour plate method as stated above.

### 2.7 Statistical Analysis

All data related to phage grafting onto cNP or bacterial capture by the probes are reported based on 10 mg of dry cNP or probes unless otherwise specified. Data are presented as mean ± standard deviation from at least three independent experiments (*n* ≥ 3). One‐way analysis of variance (ANOVA) followed by Tukey's post hoc test was used to evaluate significant differences between groups. Results with *p* < 0.05 were considered statistically significant.

## 3 Results and Discussion

### 3.1 Phage Specificity and Lytic Activity in Mixture

**FIGURE 1:**
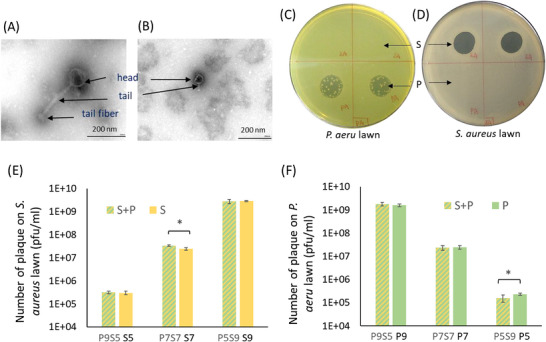
TEM micrographs of (A) S‐phage and (B) P‐phage. (C, D) Each phage formed plaques only on its respective host bacterial lawn, confirming host specificity. (E, F) Lytic activity of pure phages (S and P) and the mixed phage solution (S + P). *p* < 0.05.

The TEM micrographs show that S‐phage is approximately three times longer than P‐phage (Figure 1A,B). The head width and tail length of S‐phage measured 77 and 234 nm, respectively, whereas P‐phage had a smaller head width of 52 nm and a much shorter tail of 41 nm. S‐phage had an isometric icosahedral head and a long non‐contractile tail–it belongs to morphotype B1 of *Siphoviridae* [12]. P‐phage had an isometric icosahedral head and a short non‐contractile tail, and hence belongs to morphotype C1 of *Podoviridae* family.

The specificity assay confirmed that each phage infected only its own host strain, and neither phage infected the other bacterium (Figure 1C,D). This result indicates that only S‐phage on a probe contributes to the capture of *S. aureus*, and only P‐phage contributes to the capture of *P. aeru*.

The phage infection process begins with the recognition and attachment to specific surface receptors on the bacterial cell wall. Phages infecting Gram‐positive bacteria typically bind to peptidoglycan, teichoic acid, and lipoteichoic acids, whereas phages targeting Gram‐negative bacteria recognize lipopolysaccharides, pili, or capsules [13]. Therefore, it is highly unlikely for a phage that targets a Gram‐positive bacterium to specifically bind a Gram‐negative bacterium, or vice versa.

The results in Figure 1E,F further demonstrate that the lytic activity of each phage was unaffected by the presence of the other phage, regardless of their concentration ratio (10^4^:1, 1:1, 1:10^4^).

### 3.2 Physical and Chemical Characterization of cNP and Phage‐cNP

**FIGURE 2:**
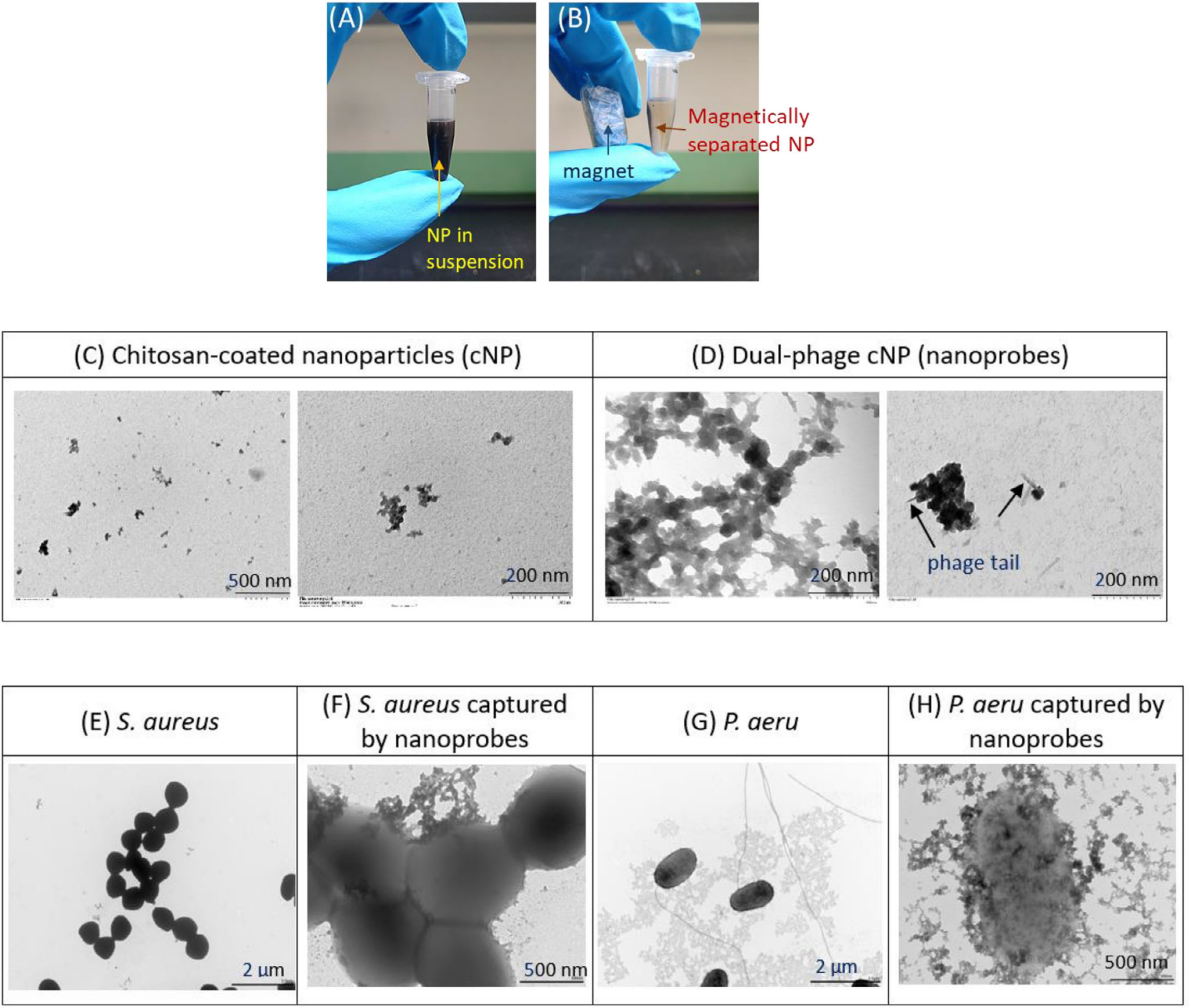
(A) cNP suspension in water. (B) Superparamagnetic cNPs were rapidly magnetized and collected using a magnet. TEM micrographs of (C) cNPs and (D) dual‐phage nanoprobes; phage tails were occasionally visible (arrows). TEM micrographs of (E) free *S. aureus* and (F) *S. aureus* captured by the S‐phage probe. TEM micrographs of (G) *P. aeru* and (H) *P. aeru* captured by the P‐phage probe.

The freshly synthesized cNPs exhibited superparamagnetic behavior and could be magnetized and collected within seconds using a neodymium magnet (Figure 2A,B). In aqueous solution, the cNPs tended to form aggregates (Figure 2C), and this aggregation became more pronounced after phage grafting (Figure 2D).

**FIGURE 3:**
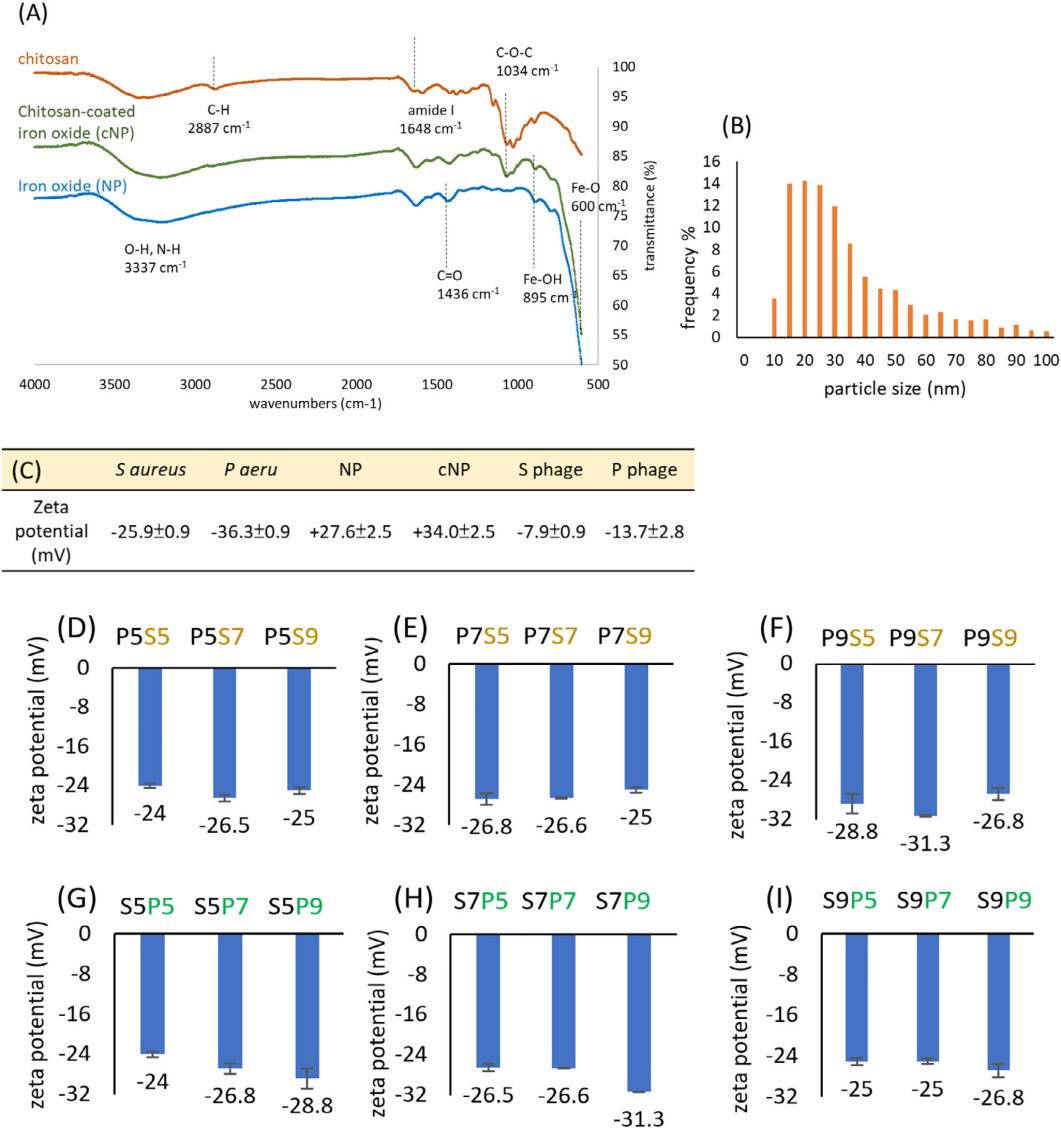
(A) FTIR spectra of chitosan, NP, and cNP. (B) Particle size distribution of cNP. (C) Zeta potentials (mV) of the two bacterial strains, NP, cNP, and the two phage strains. (D–I) Comparison of the zeta potentials of the dual‐phage nanoprobes prepared from solutions containing different phage concentrations.

The presence of the chitosan coating on the iron oxide nanoparticles was confirmed by FTIR analysis (Figure 3A). The spectrum of cNP displayed characteristic peaks for iron oxide at 569 cm^−1^ (Fe─O) and chitosan at 1024 cm^−1^ (C─O─C) and 1643 cm^−1^ (C═O) [14].

The size distribution of individual cNP particles indicated that most particles ranged from 15 to 30 nm, with approximately 58% measuring below 30 nm (Figure 3B). This nanoscale size contributes to their superparamagnetic behavior and high surface energy, which promotes aggregation in aqueous environments [15]. By having a chitosan coating on the iron oxide nanoparticles, the zeta potential of the nanoparticles went from +27 to +34 mV (Figure 3C). Iron oxide nanoparticles tend to agglomerate in order to diminish their high surface free energy. The increase in surface charge allows a stronger electrostatic repulsive force that separates the nanoparticles and reduces the size of their agglomerates [16]. This way they stay more dispersive in a solution and can more effectively capture bacterial cells. No other stabilizing agents were used for the nanoprobes because in future applications they most likely will be used as is.

As shown in Figure 3C, both bacterial strains and phages carried negative surface charges [7], while NP and cNP exhibited positive zeta potentials. The conjugation of negatively charged phages onto positively charged cNP resulted in negatively charged dual‐phage probes (Figure 3D–I). The zeta potential of the dual‐phage probes did not change significantly with increasing concentrations of S‐phage (Figure 3D–F). In contrast, the zeta potential became progressively more negative as the concentration of P‐phage increased (Figure 3G–I). This suggests that P‐phage has a more negative intrinsic surface charge than S‐phage, making the shift in surface potential more pronounced when P phage loading increases.

The specificity tests of the nanoprobes showed that the S‐probes did not capture *P. aeru* from the suspension, neither did P‐probes capture any *S. aureus*. No colonies were seen on the agar plates.

### 3.3 Single‐Phage Nanoprobes: Grafting Efficiency and Bacterial‐Capturing Performance

**FIGURE 4:**
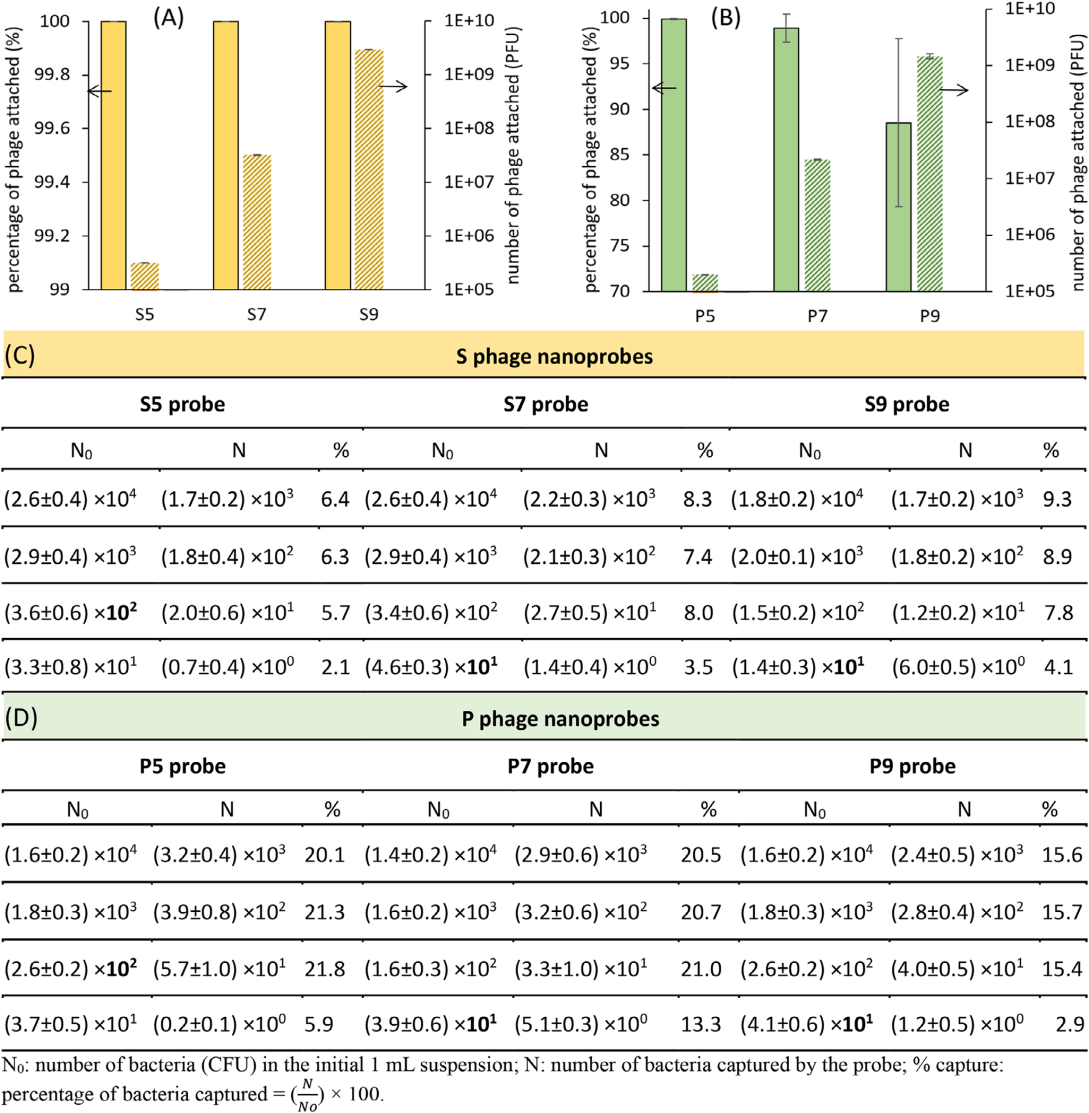
(A) Percentage and number of S‐phages in S5, S7, and S9 solutions that were grafted onto cNP. (B) Percentage and number of P‐phages in P5, P7, and P9 solutions that were grafted onto cNP. (C) Numbers and percentages of bacterial cells captured by S‐phage probes. (D) Numbers and percentages of bacterial cells captured by P‐phage probes. Bold values indicate the detection limit (PFU/mL) of each single‐phage probe.

Nearly 100% of S‐phages in the S5, S7, and S9 solutions were grafted onto cNP to form S‐phage probes (Figure 4A). Similarly, almost 100% of P‐phages in the P5 and P7 solutions were grafted onto cNP. Although only 88% of P‐phages from the P9 solution were grafted, this still resulted in more than 1 × 10^9^ PFU of P‐phages loaded onto 10 mg of cNP (Figure 4B). Overall, 10 mg of single‐phage nanoprobes carried more than 10^9^ PFU of either P‐ or S‐phage.

When single‐phage probes were added to 1 mL bacterial suspensions containing 10^1^–10^4^ CFU, the S7, P7, S9, and P9 probes consistently captured bacteria at 10^1^ CFU/mL. In contrast, S5 and P5 probes showed a higher detection limit of 10^2^ CFU/mL (Figure 4C,D).

Bacterial capture efficiency is determined by the frequency of collisions between probes and bacterial cells [17]. Because the nanoprobes could collide with only a fixed proportion of cells during the 10‐min test period, capture became nearly impossible when the bacterial concentration fell below a certain threshold.

Interestingly, the S9 and P9 probes consistently captured fewer bacterial cells than the S7 and P7 probes across all bacterial concentrations. Both the probes and bacterial cells carried negative surface charges, creating electrostatic repulsion during collision [18]. Although the intrinsic affinity between phages and their hosts typically overcomes this repulsion in nature [7], excessive phage loading (S9 and P9) may have increased electrostatic repulsion to a level that inhibited efficient phage‐bacterium binding. Zeta potentials of the probes, phages, and bacteria were measured in water to avoid ionic adsorption and desorption on sample surfaces, causing inconsistent readings. Measurements of bacterial capture were made in PBS because it has an ionic strength similar to that of a physiological environment. When the negatively charged nanoprobes and bacteria were mixed in PBS, the cations, namely Na+ wand K+, would bind the negative surfaces of the probes and the bacteria though ironic attraction. This would reduce the electrostatic repellant force between the probes and bacteria, making the binding more efficient. Zemb et al. noted that ionic strength can alter the surface charge of both phages and bacterial cells, thereby affecting adsorption behaviors [7].

Finally, the maximum capture rate for *S. aureus* was below 10% for both single‐ and dual‐phage probes, whereas capture of *P. aeru* reached 20%–30%. This difference likely reflects variations in bacterial surface receptors and phage binding affinity among species [19].

### 3.4 Dual‐Phage Nanoprobes: Phage Grafting Efficiency and Bacterial‐Capturing Performance

**FIGURE 5:**
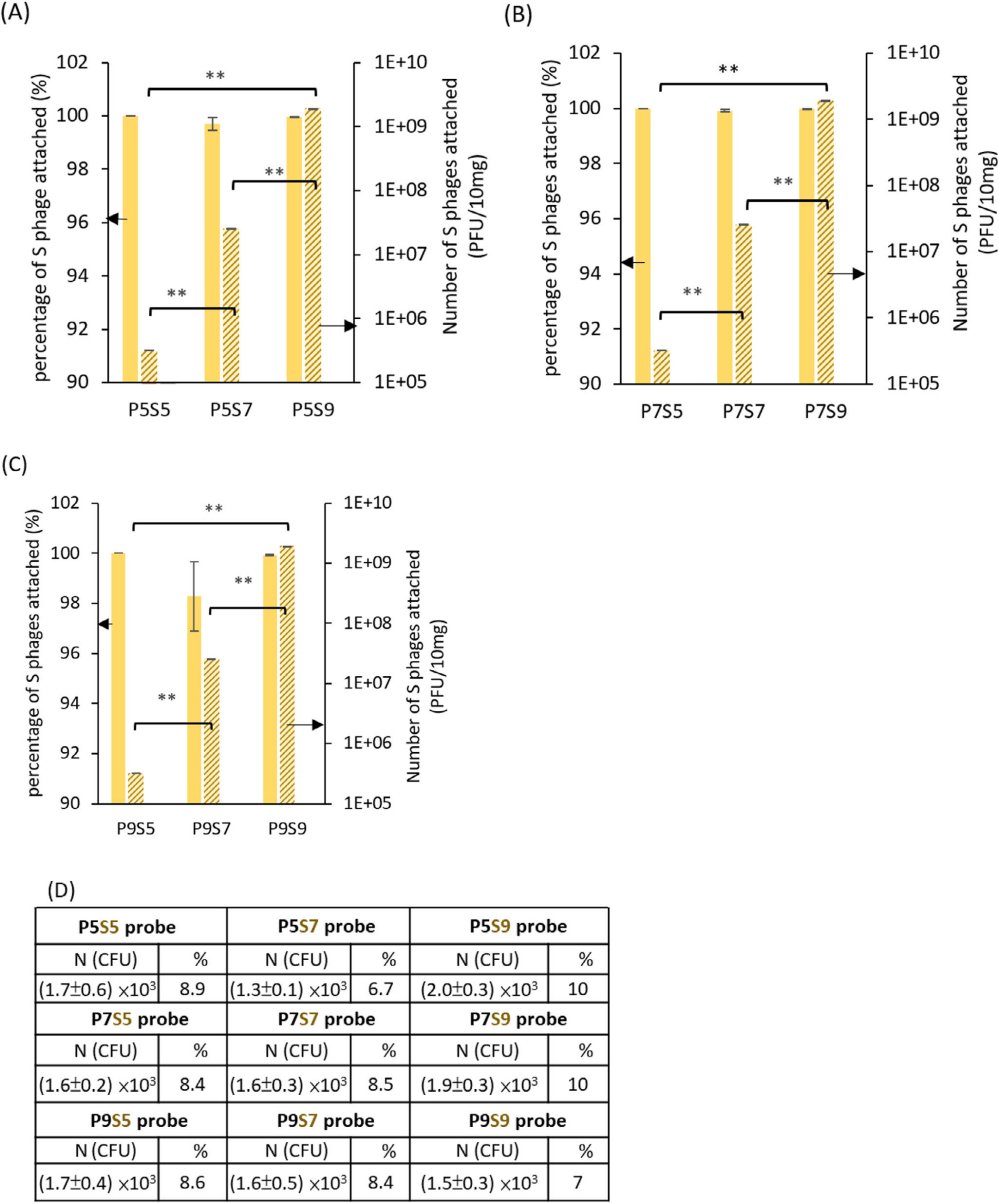
(A–C) Numbers and percentages of S‐phages in the mixed phage solutions that were grafted onto cNP. (D) Numbers and percentages of *S. aureus* captured by dual‐phage probes from 1 mL samples containing N_0_ = (1.9 ± 0.4) × 10^4^ CFU of bacterial cells. N (CFU): number of *S. aureus* captured by 10 mg of dual‐phage probe; percentage of bacteria captured = ($\frac{N}{\mathrm{No}}$) × 100%.

Regardless of the P‐phage concentration, nearly all S‐phages in the nine phage mixtures were grafted onto cNP (Figure 5A–C). In other words, the number of S‐phages on the cNP increased proportionally with the number of S‐phages in the solution.

The nine dual‐phage probes were then tested for their ability to capture *S. aureus* from 1 mL samples containing 10^4^ cells. All probes showed similar capture efficiencies, ranging from 7% to 10%, and probes with higher numbers of grafted S‐phages did not capture significantly more *S. aureus* (*p* > 0.05) (Figure 5D).

**FIGURE 6:**
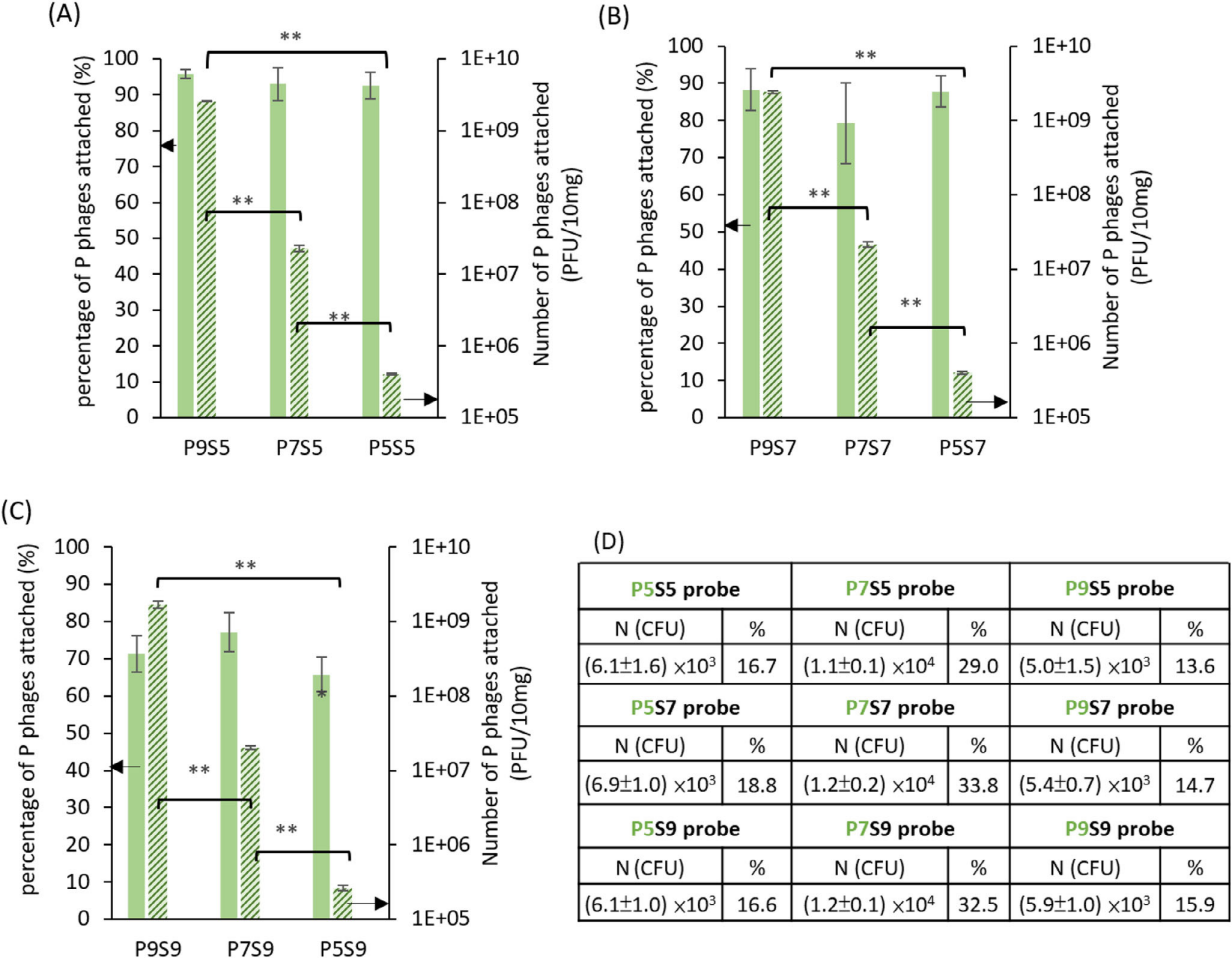
(A–C) Numbers and percentages of P phages in the mixed phage solutions that were grafted onto cNP. (D) Numbers and percentages of *P. aeru* captured by dual‐phage probes from 1 mL samples containing N_0_ = (3.7 ± 0.5) × 10^4^ CFU of bacterial cells. N (CFU): number of *P. aeru* captured by 10 mg of dual‐phage probe; percentage of bacteria captured = ($\frac{N}{\mathrm{No}}$) × 100.

The number of P‐phages grafted onto cNP increased with the P‐phage concentration in the solution, regardless of the S‐phage concentration (Figure 6A–C). At S9, however, the percentage of P‐phages grafted onto cNP decreased to 70%, suggesting that P‐phage may be less competitive than S phage in occupying surface sites on cNP. We suspect this is related to the phage size difference: when the cNP surface became crowded at high phage concentrations, the smaller S‐phage (approximately one‐third the size of P‐phage) could more easily locate available attachment sites than the larger P‐phage.

While the number of *S. aureus* captured by the dual‐phage probes did not vary with the number of S‐phages grafted, the number of *P. aeru* captured varied with the number of grafted P‐phages, though not proportionally. The P7‐series dual‐phage probes (P7S5, P7S7, and P7S9) captured significantly more *P. aeru* than the other two probe series (*p* < 0.05) (Figure 6D).

As noted earlier, an excessive number of phages on the P9‐series probes may have generated a strong negative surface potential, hindering effective interactions with the negatively‐charged bacterial cells. Therefore, a P‐phage concentration of 10^7^ PFU/mL range (P7) was optimal for preparing 10 mg of dual‐phage probes targeting *P. aeru*. Likewise, S7 probe had the lowest detection limit (Figure 4C) and S7 did not interfere with P‐phage grafting (Figure 6B), indicating that S7 was the most suitable S‐phage concentration. Overall, the S7P7 formulation produced the optimal dual‐phage nanoprobe.

The U.S. and Canadian regulations recommend fewer than 500 CFU/mL in public drinking water [20]. With a detection limit of 10^1^ CFU/mL, the S7P7 dual‐phage probe may therefore have potential applications in drinking water testing in these regions.

## 4 Conclusion

We successfully developed dual‐phage superparamagnetic nanoprobes with high sensitivity, achieving a detection limit of 10^1^ CFU/mL for both bacterial targets. The optimal probes were produced using phage concentrations of 10^7^ PFU/mL for both S‐ and P‐phages, a level readily achievable in standard laboratories. Increasing phage concentrations beyond this point led to excessive electrostatic repulsion, which impaired bacterial capture efficiency.

In this study, the P‐phage grafting efficiency decreased in the presence of high concentrations of S‐phage. These findings are specific to the phage strains used, as phage affinity, size, and surface charge vary across species. Nevertheless, isolating phages from natural environments remains a promising strategy to counter continuously evolving bacterial pathogens [21].

For practical applications, the nanoprobe system can simultaneously capture two bacterial strains, and chromogenic media may be used to differentiate colonies by color. Since phage interactions with their hosts were not affected by the presence of another phage, incorporating a third phage strain may further broaden the detection spectrum of this platform.

## Author Contributions

**Hsin‐Yi Lin**: conceptualization, methodology, formal analysis, validation, writing – review and editing, supervision, funding acquisition. **Jun‐Ping Zhang**: investigation, methodology, data curation, writing – original draft.

## Conflicts of Interest

The authors declare no conflicts of interest.

## Data Availability

The data that support the findings of this study are available from the corresponding author upon reasonable request.
